# Unfolded protein response mediator, the IRE1α-XBP1 pathway is involved in osteoblast differentiation

**DOI:** 10.1186/ar3671

**Published:** 2012-02-09

**Authors:** Takahide Tohmonda, Kazuhiro Chiba, Yoshiaki Toyama, Keisuke Horiuchi

**Affiliations:** 1 Department of Anti-aging Orthopedic Research, School of Medicine, Keio University, Tokyo, Japan; 2 Department of Orthopedic Surgery, School of Medicine, Keio University, Tokyo, Japan

## Background

To maintain the bone strength and functions, the balance between bone resorption and bone formation has to be tightly regulated. However, under certain pathological conditions, including osteoporosis and rheumatoid arthritis, the equilibrium gets disrupted, resulting in a severe bone loss. Recent studies have shown that signaling molecules involved in the unfolded protein response (UPR) are potentially involved in the coupling of bone resorption and bone formation [1-3]. In the present study, we investigated the roles of UPR mediator, the IRE1α-XBP1 pathway in osteoblast differentiation.

## Materials and methods

To induce osteoblast differentiation *in vitro*, we used recombinant human BMP-2 and mouse embryonic fibroblasts (MEFs) obtained from wild-type and *Ire1^-/- ^*embryos. Small interfering RNA-mediated gene silencing was used to suppress the expression of the target molecules of IRE1 (XBP1 and TRAF2) in wild-type MEFs. Osteoblast differentiation was evaluated by analyzing the expression levels of the transcripts for osteoblast differentiation markers (*Runx2*, *Osterix*, *Osteoclacin *and type I collagen) and alkaline-phosphatase activity.

## Results

We found that UPR is induced during osteoblast differentiation in *in vitro *and *ex vivo *experiments. Most importantly, *Ire^-/- ^*MEFs and *Xbp1*-silenced MEFs were defective in BMP2-induced osteoblast differentiation, indicating that the IRE1α-XBP1 pathway is essential for the maturation of osteoblasts. Furthermore, we found that UPR induces transcription of *Osterix *(a transcription factor indispensable for bone formation) via the IRE1α-XBP1 pathway, and that XBP1 directly binds to the promoter region of the *Osterix *gene and functions as a transcription factor. Taken together, the present study indicates that the UPR induced during osteoblast differentiation stimulates *Osterix *transcription through the IRE1α-XBP1 pathway.

## Conclusions

The present study shows that the IRE1α-XBP1 pathway is a critical component of osteoblast differentiation. Since the IRE1α-XBP1 is also involved in the production of a potent regulator for osteoclast differentiation, interferon beta [1,2], the IRE1α-XBP1 pathway may be an attractive molecular target in modulating the equilibrium between bone formation and bone resorption under pathological conditions.
